# Can the evaluation of marker placement confidence be used as an indicator of gait kinematic variability?

**DOI:** 10.3389/fresc.2023.1122303

**Published:** 2023-07-05

**Authors:** Mickael Fonseca, Xavier Gasparutto, Carcreff Lena, Gautier Grouvel, Alice Bonnefoy-Mazure, Raphaël Dumas, Stéphane Armand

**Affiliations:** ^1^ Kinesiology Laboratory, Geneva University Hospitals and University of Geneva, Geneva, Switzerland; ^2^ Univ Lyon, Univ Gustave Eiffel, Univ Claude Bernard Lyon 1, LBMC UMR_T9406, LBMC, Lyon, France

**Keywords:** marker placement, confidence, kinematics, variability, gait analysis

## Abstract

**Introduction:**

Three-dimensional gait analysis is widely used for the clinical assessment of movement disorders. However, measurement error reduces the reliability of kinematic data and consequently assessment of gait deviations. The identification of high variability is associated with low reliability and those parameters should be ignored or excluded from gait data interpretation. Moreover, marker placement error has been demonstrated to be the biggest source of variability in gait analysis and may be affected by factors intrinsic to the evaluators such as the evaluator's expertise which could be appraised through his/her experience and confidence in marker placement.

**Objectives:**

In the present study, we hypothesized that confidence in marker placement is correlated with kinematic variability and could potentially be used as part of a score of reliability. Therefore, we have proposed a questionnaire to evaluate qualitatively the confidence of evaluators in lower-limb marker placement. The primary aim of this study was to evaluate the reliability and validity of the presented questionnaire. The secondary objective was to test a possible relationship between marker placement confidence and kinematics variability.

**Methods:**

To do so, test-retest gait data were acquired from two different experimental protocols. One protocol included data from a cohort of 32 pathological and 24 asymptomatic subjects where gait analysis was repeated three times, involving two evaluators. A second protocol included data from a cohort of 8 asymptomatic adults with gait analysis repeated 12 times, per participant, and involving four evaluators with a wider range of experience.

**Results:**

Results demonstrated that the questionnaire proposed is valid and reliable to evaluate qualitatively the confidence of evaluators in placing markers. Indeed, confidence scores were correlated with the actual variability of marker placement and revealed the evaluator's experience and the subjects' characteristics. However, no correlation was observed between confidence scores and kinematic variability and the formulated hypothesis was not supported.

## 1. Introduction

Three-dimensional gait analysis (3DGA) is widely used in the assessment of motor disorders and to support treatment decision-making. Variability in 3DGA is due to a combination of intrinsic and extrinsic factors. Intrinsic factors refer to the natural variability associated with the capacity of a subject to repeat the same gait movement across cycles, within or between days, and it is considered an indicator of gait impairments, typically described as intrinsic variability (1). On the other hand, extrinsic factors are associated with measurement error and are caused by a combination of parameters such as marker placement, instrumentation, soft tissue artifacts, and data processing (2–5). Variability associated with extrinsic factors reduces confidence reliability of the measure and the interpretation. However, several studies reported variability within the measured data by reproducing gait data collection under the same conditions (6–9). Among the complete set of data measured, kinematic parameters are the most variable, with highest level of variability observed in the transversal plane such as the hip rotation (10). In addition, extrinsic variability has been demonstrated to be generally higher than intrinsic variability (6). With respect to extrinsic variability, marker placement has been reported as the biggest source of variability in 3DGA (11). Marker placement relies on the correct palpation and identification of the subcutaneous Anatomical Landmarks (AL) and its precision and accuracy are sometimes difficulted by their large and curvy characteristics (4). The correct identification of ALs depends on the expertise of the evaluator, allied with the anatomy of the subject since underlying adipose tissue or bony deformations may render difficult the palpation or correct positioning of the markers. For instance, a subject with high subcutaneous adipose tissue has been proven to be associated with higher difficulty in palpation (12, 13). Even if the AL is correctly identified for this subject, the accurate location of the skin marker will probably be reduced and consequently affects the definition of the segment coordinate systems. Therefore, we can expect that the difficulties encountered by the evaluator to place the marker (correct identification of the AL and presence of soft tissues) will impact his/her confidence in this placement. This confidence in marker placement and the experience of the evaluator’s can be considered surrogate measures of his/her expertise.

The application of 3DGA in clinics require a reliable and accurate measurement setup, including the placement of markers by the evaluator. Contrarily to the reliability, there is no demonstrated way to measure the accuracy of kinematic data. The most commonly applied biomechanical model in 3DGA is known as the Conventional Gait Model (CGM) and it has been proven to be highly sensitive to marker placement accuracy, and thus dependent on the expertise of the evaluator (14–16). The CGM may be applied under different variants. The most commonly applied version of CGM in 3DGA is its basic version, composed of seven segments, for the lower limb evaluation, and a set of twenty-two reflective markers (17). Therefore, the question of whether an evaluator should be well prepared and experienced to place markers in a gait analysis session was debated. Previous results have shown that the more experienced the experimenter, the greater the repeatability of marker placement (18). Thus, suitable training has proven to play a more important role than experience in gait analysis. However, the evaluation was performed between only two evaluators (one experienced and one novice) in a short sample size (10 asymptomatic subjects) and each evaluator collected one gait measurement session per subject. In addition, the level and heterogeneity of the population observed in terms of BMI were low. Thus, due to these factors, the data collected in this previous study may not be sufficient for evaluating possible statistically significant differences between the evaluators and between the subjects.

Extrinsic variability is inherent to measuring gait data and negatively affects the assessment of gait deviations during the interpretation of the results (6). Differences in data concerning the normative reference database are required to be higher than the estimated variability to be accepted as true gait deviations. Being so, the estimation of such variability could be important to increase or reduce the reliability of kinematic data.

Therefore, we hypothesized that the confidence of evaluators in placing markers may be related to measurement error in gait kinematics data and have the potential to be used as an indicator of joint (hip, knee, ankle) and segment (pelvis, foot) angle variability. Typically, a very low confidence in marker placement can flag the kinematics as unreliable. Thus, the first objective of this study is to evaluate the reliability and validity of a proposed custom-made questionnaire for reporting qualitatively the Confidence in Marker Placement (CMP) from the evaluators. To do so, we intended to evaluate the relationships between CMP scores relative to other aspects of measurement gait such as the evaluator’s experience, subject characteristics, and marker placement precision, and to characterize its distribution. The second objective is to evaluate the correlation between CMP scores and kinematic variability.

## 2. Methods

**Figure 1:**
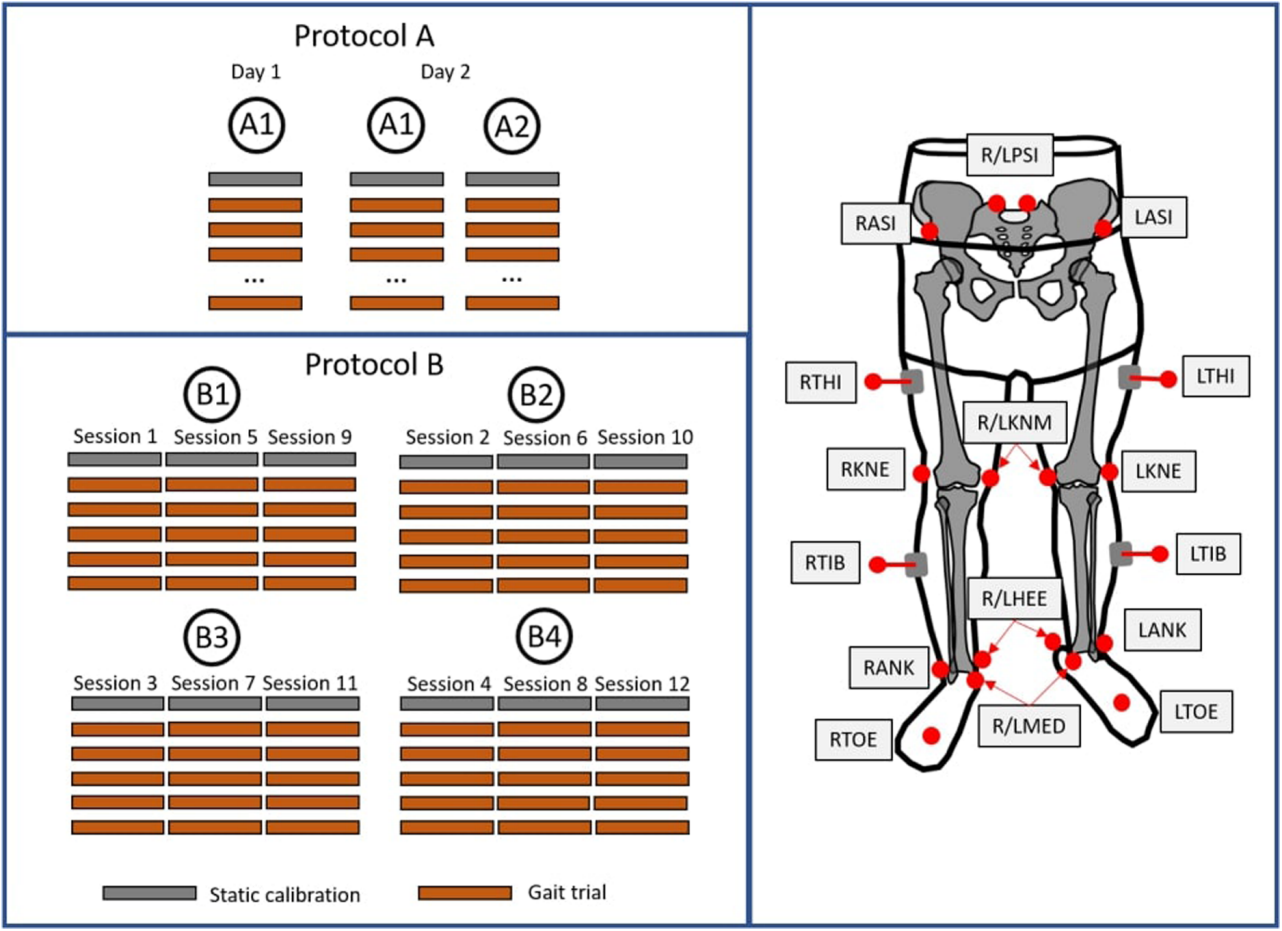
Illustration of the two protocols applied in the present study (left) and marker placement protocol applied for both protocols (right).

Two test-retest experimental protocols were used (Figure 1). Firstly, we used an experimental protocol (A) with a test-retest methodology on a heterogeneous cohort, incorporating asymptomatic subjects and patients with motor disorders within different age groups. Data collection was repeated three times, for each subject among two evaluators. Secondly, we have defined an experimental protocol (B) composed of a test-retest methodology and including four evaluators with different levels of experience in marker placement. This protocol involved the participation of eight asymptomatic adults, and for each, data collection was repeated twelve times within a unique visit (three sessions per evaluator).

### 2.1. Subject populations

Protocol A involved the recruitment of 56 subjects, including 24 asymptomatic participants, [mean (SD) age: 18.3 (9.6) years; height: 155.4 (21.7) cm; mass: 52.1 (19.2) kg; 12 males and 12 females] and 32 patients [24 patients with CP and 8 patients with other motor disorders; mean (SD) age: 18.4 (9.7) years; height: 156.7 (17.5) cm; mass: 52.4 (19.2) kg; 25 males and 7 females]. In protocol B, 8 asymptomatic adults were recruited [mean (SD) age; 31.2 (11.0) years; height: 171.2 (8.9) cm; mass: 71.5 (16.1) kg; 4 males and 4 females] with no pathological condition affecting normal motor ability. These protocols were approved by the “Commission Cantonale d’Éthique de la Recherche de Genève” (CCER-2020-00358) and all subjects provided written informed consent (legal tutors signed the consent for non-adult subjects). The exclusion criteria for all groups were known pregnancy and no allergy to adhesive tape.

### 2.2. Experimental protocol A

In protocol A, subjects visited the laboratory on two occasions 10 days apart. Two evaluators with required training for AL identification were responsible for conducting the complete gait analysis sessions. Evaluators A1 and A2 have approximately four and two years of experience in gait analysis, respectively. On the first visit, evaluator A1 was responsible for placing the markers and each participant performed one gait analysis session, including one static and a minimum of ten gait trials in the 10 m walkway, barefoot and containing at least one gait cycle per trial. On the second visit, the subjects were asked to repeat two gait analysis sessions, conducted by evaluators A1 and A2, respectively. Reflective markers (14 mm) were placed following the Conventional Gait Model described in (19) and palpation followed the guidelines previously described (20) (description of marker locations in Supplementary Information S1). A 12-camera motion capture system (Oqus7+, Qualisys, Göteborg, Sweden) tracked the marker trajectories at 100 Hz.

### 2.3. Experimental protocol B

In protocol B, the subjects visited the laboratory on one occasion. Four evaluators were responsible for conducting three different marker placement sessions each. All evaluators were properly trained and differed in the level of experience: evaluator B1 with more than ten years of experience in clinical practice, with over a hundred gait analysis sessions per year; evaluators B2 and B3, have approximately four and two years of experience in gait analysis, respectively, with approximately fifty sessions per year; evaluator B4 had no previous experience in gait analysis. Reflective markers were placed following the same biomechanical model applied for protocol A. In addition, clusters of markers were added during the entire set of data acquisition in the pelvis and each of the lower limb segments to standardize the reference segment position and orientation. Moreover, the same equipment was used for both protocols.

### 2.4. Marker placement confidence questionnaire

A custom-made questionnaire was designed to report qualitatively the confidence of evaluators in placing the markers (See Supplementary Information S2). For each marker, a scale of confidence is provided ranging from zero (extremely low confidence) to ten (extremely confident). Evaluators of each protocol filled out the questionnaire after each marker placement session.

### 2.5. Statistical analysis

Firstly, following the COSMIN guidelines for assessing the methodological quality of the measurement of CMP scores, reliability and validity were evaluated (21). To answer this first objective, the reliability of the CMP scores was evaluated in both protocols A and B using the Interclass Correlation (ICC) (3,1) (22), typically used in agreement studies with interval ratings (23).

Several relationships were then evaluated to analyze the validity of the CMP score. The statistical differences in CMP scores between the two populations (asymptomatic and pathologic) were tested with protocol A. Additionally, with protocol A, Spearman rank correlation coefficients between CMP scores among all markers were calculated, with alpha values of *p* < 0.05 regarded as significant. Markers were grouped by segments and correlations among the groups and between CMP scores and subject’s characteristics such as body mass, BMI, pelvis width, leg length, and age were analyzed. The statistical differences in CMP scores between the four evaluators were tested with protocol B for all markers and groups of markers. The validity of CMP was also evaluated relative to the marker precision estimation provided by protocol B with the Spearman rank correlations between the CMP score and marker precision, for each marker. In this analysis of the CMP score validity, statistical differences were tested and correlations were analyzed by Spearman rank correlation coefficients, with *p* < 0.05 considered as significant. The calculation of marker placement precision relied on the set of clusters of markers. Marker locations were computed with respect to the respective cluster coordinate system for standardization. Therefore, marker placement precision for each session was calculated as the difference in the positioning of the markers concerning the mean location among all twelve sessions for each subject of the corresponding markers.

**Figure 2:**
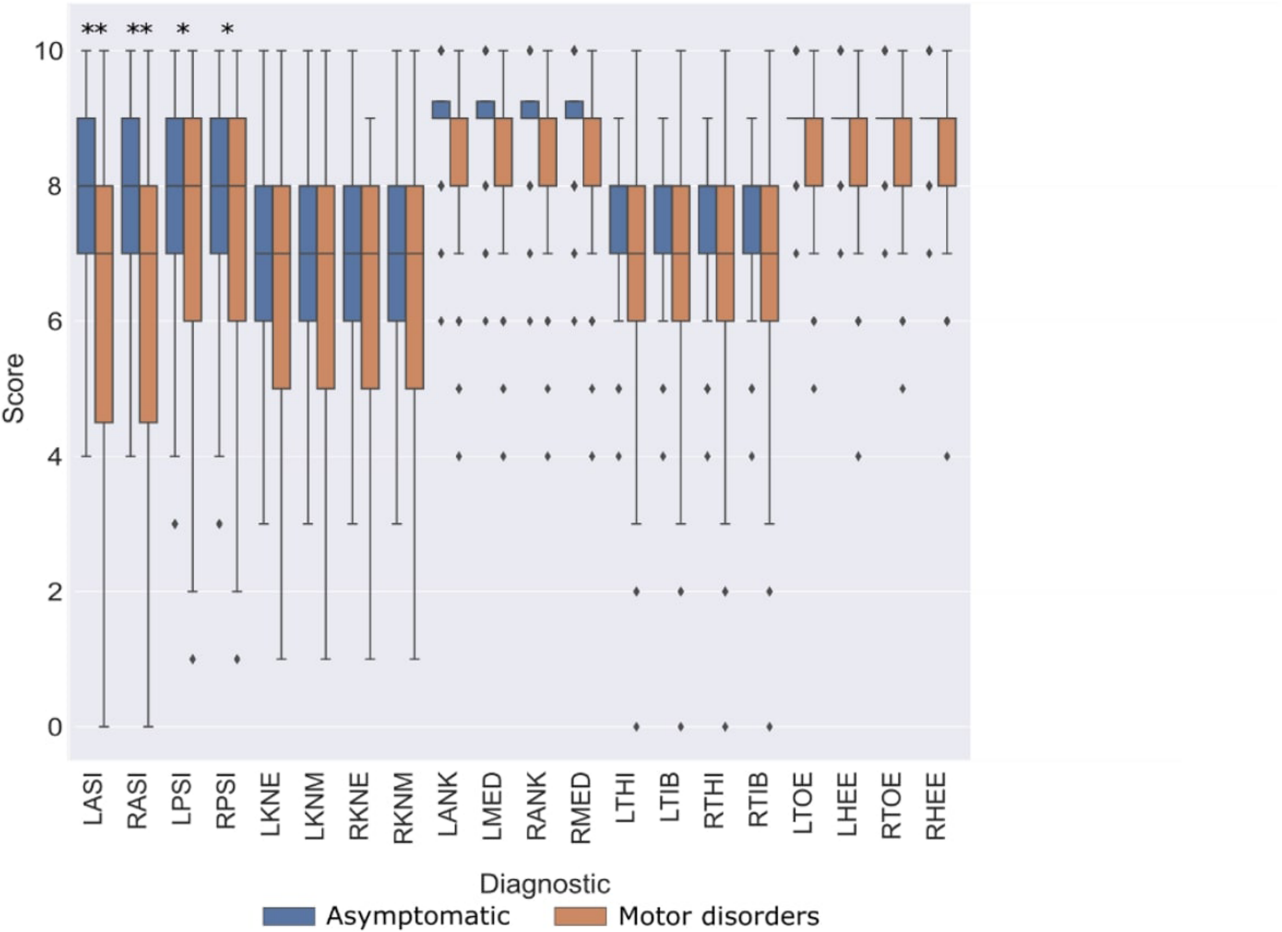
Diagram describing the relationships evaluated and experimental protocols (**A,B**) used for each relationship.

To answer the second objective, the correlation between mean CMP scores per group of markers and inter-session kinematic variability was evaluated with protocol A (Figure 2). Kinematic data were calculated using the PyCGM2 open-source library (https://github.com/pyCGM2/pyCGM2) (19). The 11 calculated segment/joint angles were pelvis tilt, obliquity and rotation, hip flexion/extension, adduction/abduction and rotation, knee flexion/extension, varus/valgus and rotation, ankle flexion/extension and foot progression. A Spearman rank correlation coefficient was applied, with *p* < 0.05 considered statistically significant. Intra-evaluator kinematic variability associated with protocol A was calculated as the standard deviation of the mean kinematics acquired among the three sessions of each participant.

## 3. Results

The diagram represented in Figure 2 illustrates the relationships evaluated and the protocols used.

### 3.1. Reliability of CMP scores

The reliability evaluation, provided in Table 1, reported the ICC calculated among the CMP scores for the data of the two protocols separately. Very similar values were obtained between both lower limb sides. On the one hand, protocol A demonstrated high reliability (ICC ≥ 0.75) for the CMP of pelvic markers and moderate reliability (0.75 >ICC ≥ 0.5) for the remaining markers. On the other hand, CMP for all markers resulted in high reliability (ICC > 0.75) on protocol B.

**Table 1:** Inter-evaluator reliability (ICC) for CMP scores compared between both protocols.

Marker	Protocol A	Protocol B	Marker	Protocol A	Protocol B
LASI	0.90	0.85	RASI	0.90	0.82
LPSI	0.84	0.82	RPSI	0.84	0.83
LTHI	0.63	0.76	RTHI	0.70	0.77
LKNE	0.74	0.83	RKNE	0.76	0.83
LKNM	0.74	0.84	RKNM	0.76	0.84
LTIB	0.64	0.75	RTIB	0.71	0.75
LANK	0.59	0.77	RANK	0.59	0.76
LMED	0.59	0.76	RMED	0.59	0.76
LHEE	0.66	0.77	RHEE	0.66	0.78
LTOE	0.57	0.84	RTOE	0.57	0.84

### 3.2. Validity of CMP scores

**Figure 3:**
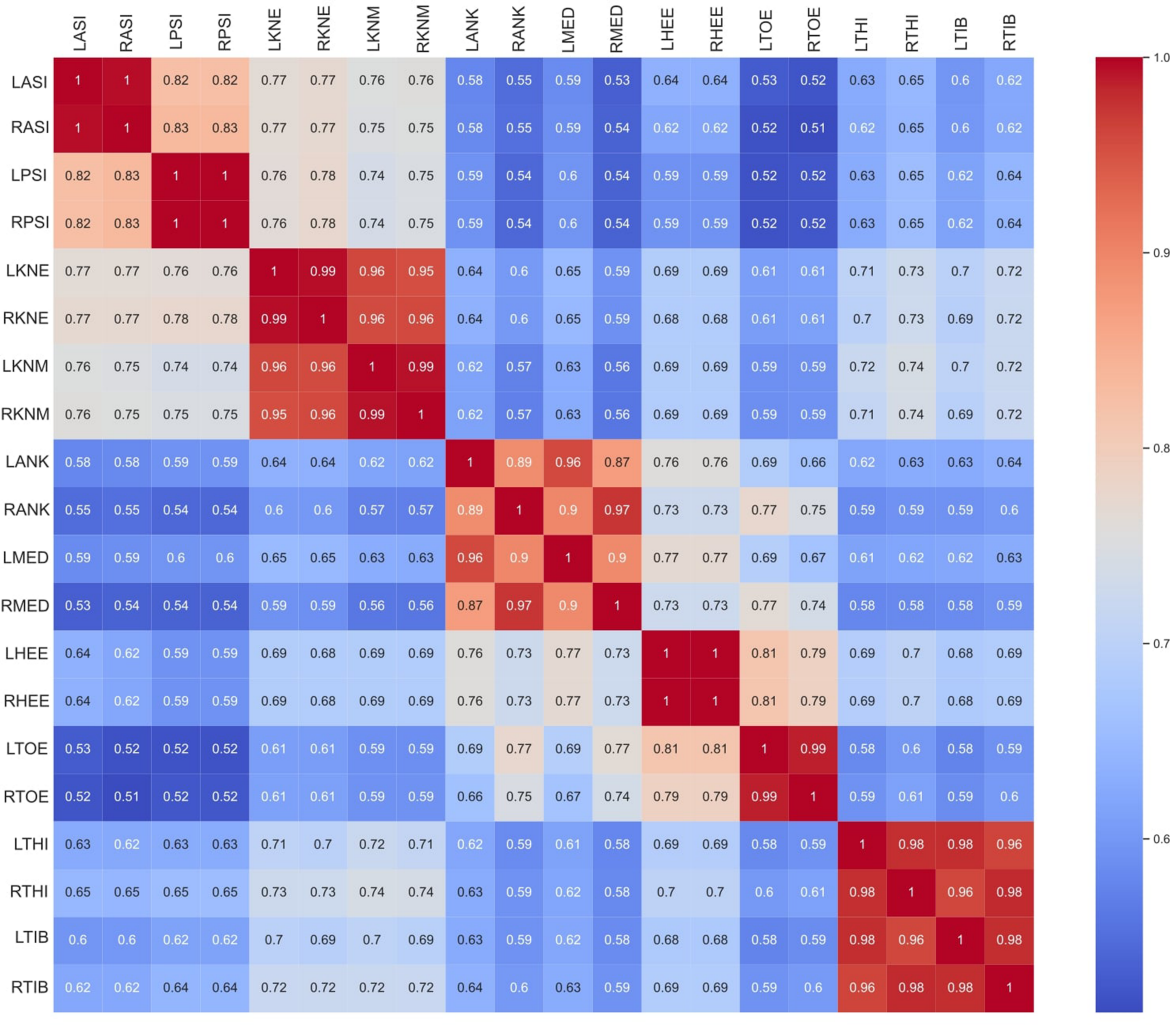
Comparison of CMP scores between asymptomatic subjects (blue) and patients with motor disorders (orange). Statistically significant differences among populations (*p* < 0.05 marked with “*”, and *p* < 0.005 marked with “**”).

Figure 3 reports the distribution of CMP scores through the different markers among both populations in protocol A. CMP score is observed widely variable across the pelvis, femoral epicondyles, and wands. It also shows a higher variance of CMP scores associated with patients, compared to the asymptomatic participants with significant differences reported. On the other hand, very low variability was observed in the CMP score of the tibial malleolus and foot markers among all subjects from both populations.

**Figure 4:**
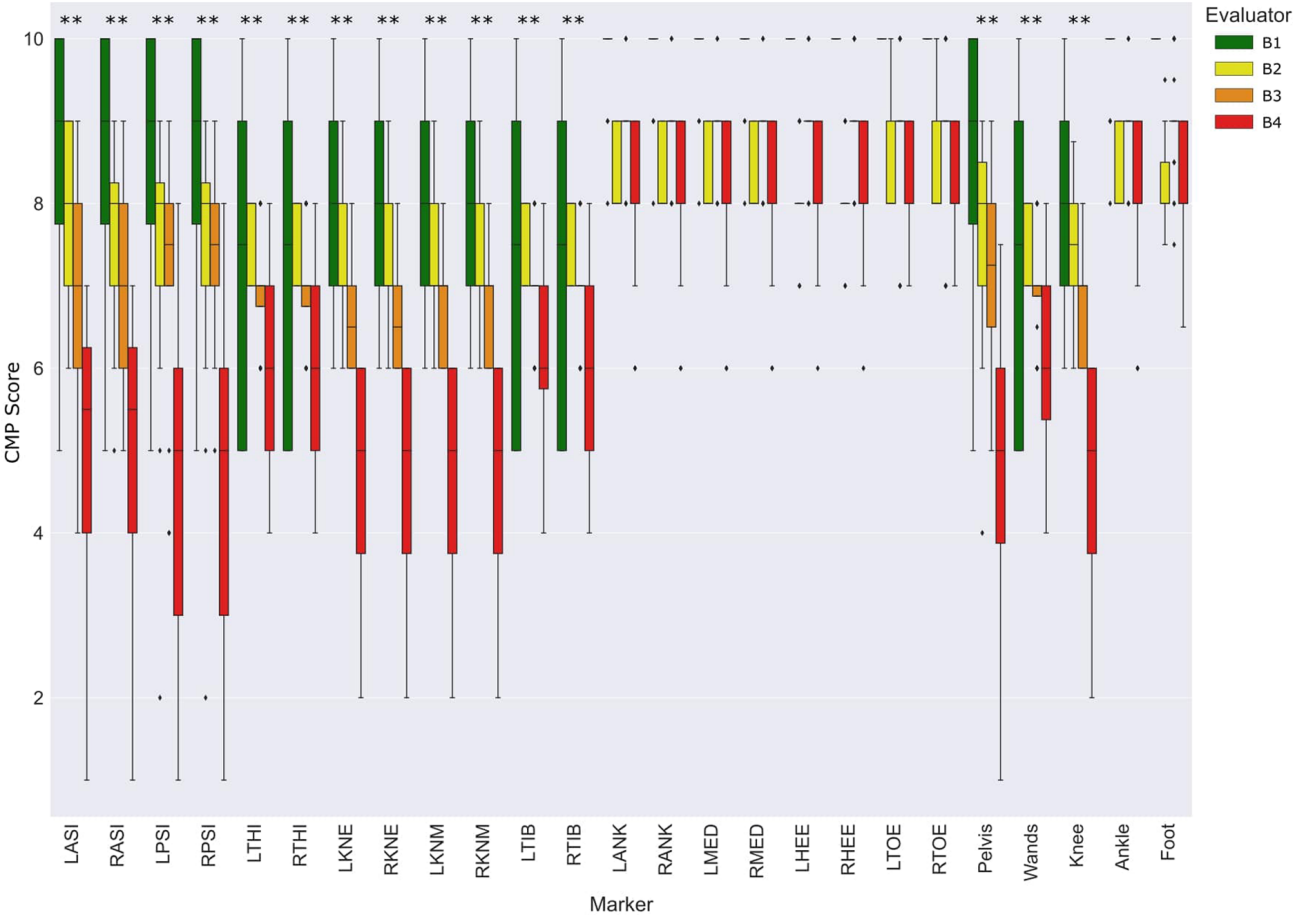
Correlation of CMP scores among markers.

**Figure 5:**
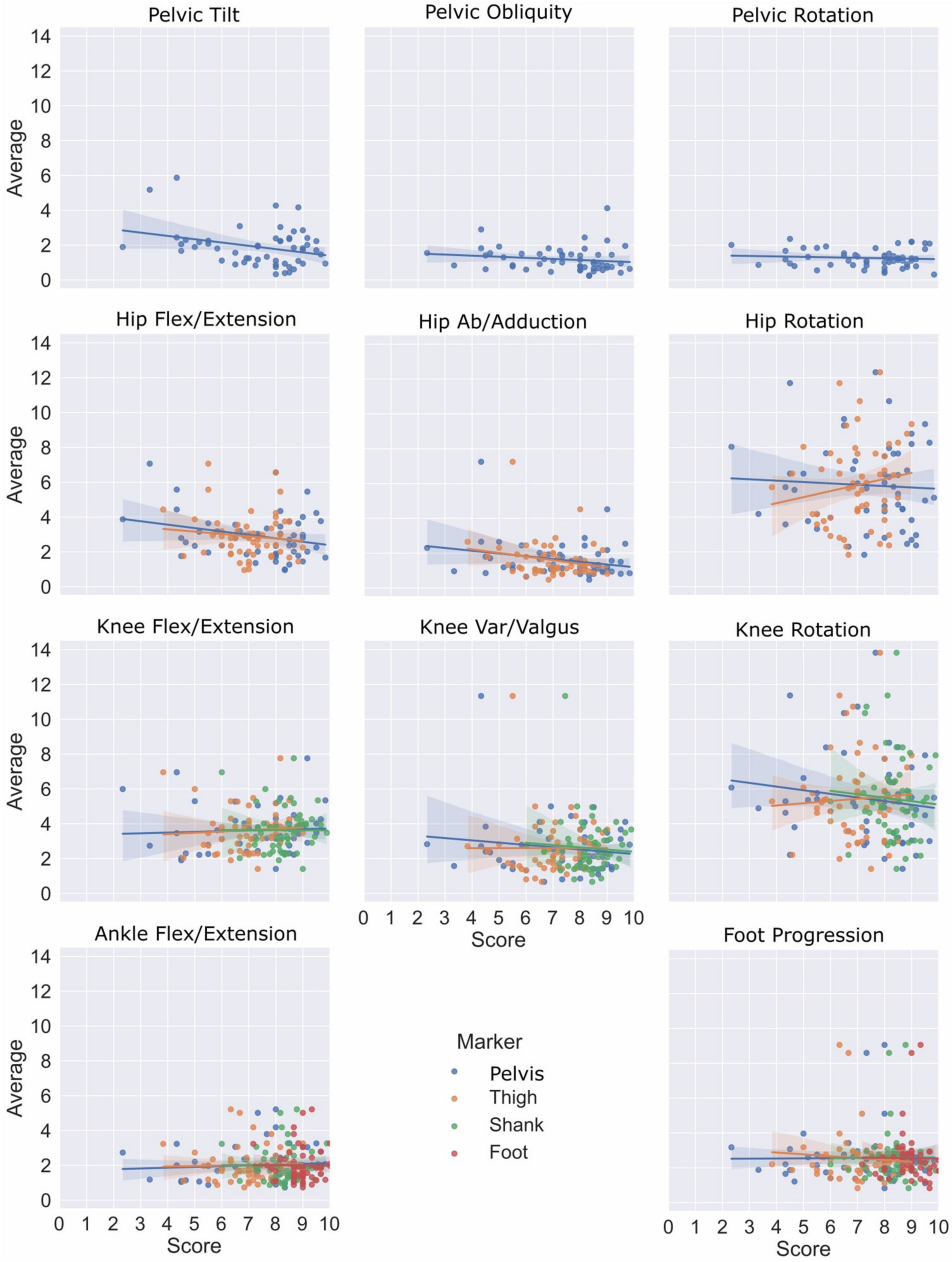
Correlation heatmap between grouped CMP scores with kinematic variability, subject’s characteristics and the same CMP scores.

The correlation between CMP scores among all markers is reported in Figure 4. CMP scores among markers are extremely correlated within the matching contra-lateral markers (i.e., LASI and RASI). Additionally, the results show that evaluators tend to be equally confident among groups of markers of the same typology (wands) and segment (pelvis, thigh, shank, and foot). Thus, a subset of markers was grouped as follows: Pelvis (L/RASI and L/RPSI); Knee (L/RKNE and L/RKNM); Ankle (L/RANK and L/RMED); and Foot (L/RHEE and L/RTOE); Wand (L/RTHI and L/RTIB). Correlations between CMP scores among groups of markers and between CMP scores and the subject’s characteristics are presented in Figure 5. Some subjects’ characteristics, such as BMI showed a good correlation with CMP scores of pelvic and thigh markers.

**Figure 6:**
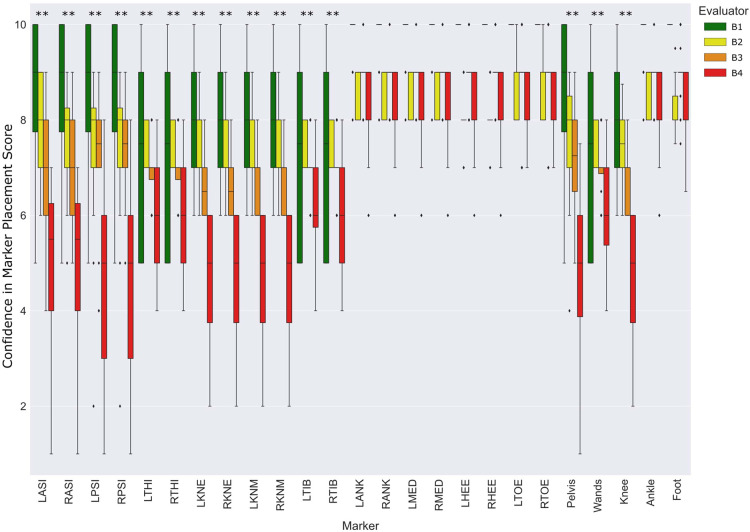
Distribution of CMP scores per evaluator and marker among the entire population relative to protocol B. Statistically significant differences among evaluators (*p* < 0.05 marked with *, and *p* < 0.005 marked with **). The absence of colored boxes from the boxplot represents an IQR equal to the median, due to a very low variance of CMP scores estimated on that specific marker.

The distribution of CMP scores for each marker and groups of markers among evaluators in protocol B is presented in Figure 6. The CMP scores for pelvic, thigh, shank, and wand groups of markers were significantly different according to the experience level of the evaluators. Similarly, to the results observed in Figure 3, ankle and foot markers showed very low variance while the remaining markers resulted in a wide range of CMP scores. Moreover, Table 2 represents the correlation analysis between the CMP scores of each marker with the precision, decomposed per direction (medial-lateral, anterior-posterior and proximal-distal) of the respective marker per session. Moderate correlations, with statistical significance, were observed for all pelvic, femoral, and wand markers in at least one of the directions.

**Table 2:** Spearman’s rank correlation between CMP scores and respective marker precision, in three directions.

Direction	Anterior-Posterior		Medial-Lateral		Proximal-Distal	
	*R*	*p*	*R*	*p*	*R*	*p*
Marker						
LASI	0.060	0.563	−0.411	**0.000**	−0.135	0.189
RASI	0.032	0.757	−0.369	**0**.**008**	−0.077	0.458
LPSI	−0.285	**0**.**005**	−0.399	**0**.**000**	−0.094	0.362
RPSI	−0.315	**0**.**002**	−0.359	**0**.**022**	−0.161	0.118
LTHI	−0.145	0.158	−0.316	**0**.**002**	−0.418	**0**.**000**
RTHI	−0.155	0.131	−0.177	0.084	−0.460	**0**.**000**
LKNE	−0.266	**0**.**009**	−0.105	0.307	−0.429	**0**.**000**
RKNE	−0.194	**0**.**041**	−0.039	0.709	−0.509	**0**.**000**
LKNM	0.048	0.639	0.055	0.596	−0.422	**0**.**000**
RKNM	0.059	0.570	0.041	0.695	−0.412	**0**.**000**
LTIB	0.069	0.506	−0.212	**0**.**039**	−0.179	0.081
RTIB	−0.011	0.918	−0.226	**0**.**027**	−0.326	**0**.**001**
LANK	−0.121	0.140	−0.062	0.546	−0.161	0.116
RANK	−0.369	**0**.**000**	−0.116	0.259	0.164	0.110
LMED	−0.259	**0**.**011**	−0.146	0.157	−0.024	0.820
RMED	−0.208	0.059	0.073	0.482	0.031	0.764
LHEE	0.127	0.218	0.086	0.404	−0.164	0.110
RHEE	0.126	0.220	−0.143	0.165	0.006	0.951
LTOE	−0.334	**0**.**001**	−0.292	**0**.**004**	0.000	0.997
RTOE	−0.230	**0**.**024**	−0.310	**0**.**002**	0.076	0.064

Bold values for *p*-value under <0.05.

### 3.3. CMP vs. kinematics variability

**Figure 7:**
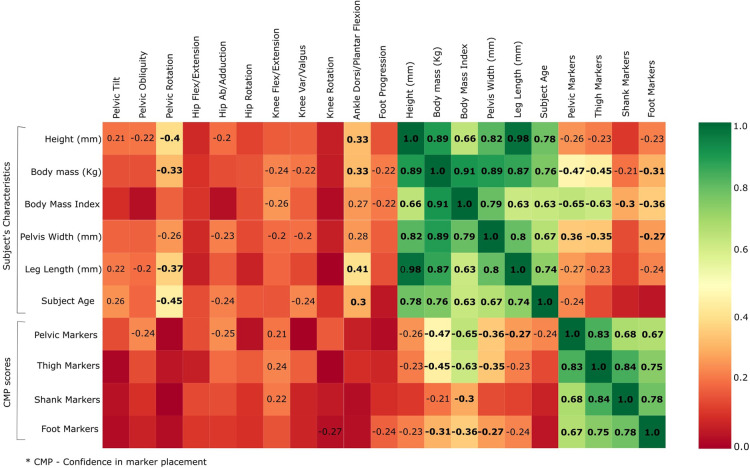
Relationship between inter-session kinematic variability and mean of CMP scores by groups of markers.

The results reported in Figure 7, with linear regression, demonstrate a low correlation between CMP scores (grouped markers) and inter-session kinematics variability in protocol A. Considering the top-down architecture of the CGM, markers of segments that are not used to calculate specific joint kinematics were not presented (i.e., foot markers do not affect the calculation of hip kinematics). Moreover, the heatmap represented in Figure 5 quantifies the correlation between the inter-session variability, the CMP scores (grouped markers) and, as mentioned before, the subject’s characteristics. However, none of those parameters, although related altogether (e.g., thigh markers with the pelvis and shank markers, pelvis markers with BMI) demonstrated a good correlation with inter-session variability.

## 4. Discussion

In the present study, a questionnaire has been proposed to evaluate qualitatively the relevance of the evaluator’s confidence in marker placement. The first aim was to evaluate the reliability and validity of CMP scores. The reliability of the proposed questionnaire was evaluated with ICC (Table 1) and showed good to moderate reliability for all markers. Moreover, the lower reliability observed in the tibial and foot skin markers may be explained by the low variance observed in CMP for those markers. Thus, it is important to take into consideration the dispersion of the rating samples when interpreting ICC values (24). On the other hand, to evaluate its validity, we have analyzed how well the CMP scores transmit qualitatively the sensation of confidence from evaluators in placing markers on the lower limbs. The distribution of the CMP scores (Figures 3, 4) demonstrated that the confidence related to the placement of some markers (pelvic, femoral, and wands) varies widely among subjects while others (tibial and foot) showed constant high confidence and with low variance. Lower confidence in the placement of pelvic, femoral, and wand markers in the pathological in comparison with the asymptomatic group was observed (Figure 3) with significant differences between the populations for the pelvis markers. This may explain the higher variability observed for pathological subjects in the literature (25). In addition, significant differences between the confidence reported for those markers with the experience of the evaluator were observed (Figure 6). A significant correlation was also observed between CMP scores among skin markers located within the same segment (i.e., pelvis) and among the wands (Figure 4). In addition, CMP scores showed a significant correlation with BMI. Thus, it suggests that underlying adipose tissue negatively affects the palpation of anatomical landmarks, especially on the pelvis and thigh markers, and consequently may be the cause of previously reported reduction of marker placement precision for subjects with higher BMI (13). Finally, confidence was significantly correlated with marker placement precision in at least one direction, especially for the pelvic and femoral markers (Table 2). All these results suggest that the CMP scores can robustly reflect the difficulties to place markers on the pelvis and thigh segments of a specific population with pathology or more adipose tissues, especially for less experienced evaluators. These perceived difficulties, quantified by the questionnaire, are related to the actual marker precision. It is important to note that marker misplacement follows mostly a bi-planar direction (i.e., pelvic markers are misplaced mainly in the anterior-posterior or proximal-distal directions). This may naturally explain why the correlation between CMP scores and marker placement precision (Table 2) is not found significant in one of the three directions for each marker.

The second goal of the study was to evaluate the correlation between the marker placement confidence reported subjectively with the output kinematics variability measured by test-retest. Considering the good correlation among markers previously described (Figure 4), we have considered the mean of correlated markers for simplification to evaluate the CMP scores with kinematics variability. Thus, the mean CMP scores reported on the markers of the pelvis, thigh, shank, foot, and wands were used, and no significant correlation has been observed. This observation may be explained by the complexity of the effect of marker placement on kinematics. As previously reported by another study with the CGM (26), the impact of one marker misplaced can be enhanced or mitigated by the misplacement of another marker. Moreover, while low confidence would be undoubtedly related to low reliability, high confidence would not be systematically related to high reliability.

In conclusion, the proposed questionnaire to evaluate marker placement confidence has been demonstrated to be valid and reliable. However, no significant correlation has been observed between confidence scores and kinematics variability in the specific case of CGM. The proposed questionnaire may be useful in a research context to test other gait analysis protocols and models from the perspective of managing uncertainty in the clinical assessment of movement disorders.

## Data Availability

The raw data supporting the conclusions of this article will be made available by the authors, without undue reservation.
