# Supplementary material for: Can the evaluation of marker placement confidence be used as an indicator of gait kinematic variability?
Source: Front Rehabil Sci. 2023 Jul 5;4:1122303. doi: 10.3389/fresc.2023.1122303 (PMC10368473; doi:10.3389/fresc.2023.1122303)
Supplement: Supplementary file 1 [file Datasheet1.pdf]

Supplementary Material

1 Marker set-up information

Table 1. Palpation guidelines and marker name list

Marker	Location
(L/R)ASI	Left/right anterior superior iliac spine
(L/R)PSI	Left/right posterior superior iliac spine
(L/R)THI	Over the lower lateral 1/3 surface of the left/right thigh
(L/R)KNE	On the flexion-extension axis of the left/right knee
(L/R)KNM	Left/right medial femoral epicondyle
(L/R)TIB	Over the upper lateral 1/3 surface of the left/right thigh
(L/R)ANK	On the lateral malleolus along an imaginary line that passes through the left/right transmalleolar axis
(L/R)MED	Left/right edial tibial malleolus
(L/R)HEE	On the left/right calcaneus at the same height above the plantar surface of the foot as the toe marker
(L/R)TOE	Over the second metatarsal head, on the mid-foot side of the equinus break between fore-foot and mid-foot.

2 Questionnaire of marker placement confidence

Table 2. Illustration of the proposed questionnaire for measuring the marker placement confidence applied during the experimental protocols.

PROJECT VAR
Participant Reported Outcome Measures - PROMs

A remplir par les HUG
Participant n°:
Session n°:
Digitalised : ☐ (on __/__/20__ by :)

VAS – Feedback concerning confidence in marker placement

Participant:______________________ Tester:________________________ Date: _______________

Low confidence ⟶ High confidence

Cause of low confidence (if applicable):

													Palpation	Soft tissue
PELVIS	LASI	0	1	2	3	4	5	6	7	8	9	10	☐	☐
	RASI	0	1	2	3	4	5	6	7	8	9	10	☐	☐
	LPSI	0	1	2	3	4	5	6	7	8	9	10	☐	☐
	RPSI	0	1	2	3	4	5	6	7	8	9	10	☐	☐
FEMUR	LKNE	0	1	2	3	4	5	6	7	8	9	10	☐	☐
	LKNM	0	1	2	3	4	5	6	7	8	9	10	☐	☐
	RKNE	0	1	2	3	4	5	6	7	8	9	10	☐	☐
	RKNM	0	1	2	3	4	5	6	7	8	9	10	☐	☐
TIBIA	LANK	0	1	2	3	4	5	6	7	8	9	10	☐	☐
	LMED	0	1	2	3	4	5	6	7	8	9	10	☐	☐
	RANK	0	1	2	3	4	5	6	7	8	9	10	☐	☐
	RMED	0	1	2	3	4	5	6	7	8	9	10	☐	☐
WAND	LTHI	0	1	2	3	4	5	6	7	8	9	10	☐	☐
	LTIB	0	1	2	3	4	5	6	7	8	9	10	☐	☐
	RTHI	0	1	2	3	4	5	6	7	8	9	10	☐	☐
	RTIB	0	1	2	3	4	5	6	7	8	9	10	☐	☐
FOOT	LTOE	0	1	2	3	4	5	6	7	8	9	10	☐	☐
	LHEE	0	1	2	3	4	5	6	7	8	9	10	☐	☐
	RTOE	0	1	2	3	4	5	6	7	8	9	10	☐	☐
	RHEE	0	1	2	3	4	5	6	7	8	9	10	☐	☐
